# The #HOPE4LIVER Single-Arm Pivotal Trial for Histotripsy of Primary and Metastatic Liver Tumors

**DOI:** 10.1148/radiol.233051

**Published:** 2024-09-03

**Authors:** Mishal Mendiratta-Lala, Philipp Wiggermann, Maciej Pech, Xavier Serres-Créixams, Sarah B. White, Clifford Davis, Osman Ahmed, Neehar D. Parikh, Mathis Planert, Maximilian Thormann, Zhen Xu, Zachary Collins, Govindarajan Narayanan, Guido Torzilli, Clifford Cho, Peter Littler, Tze Min Wah, Luigi Solbiati, Timothy J. Ziemlewicz

**Affiliations:** From the Department of Radiology, University of Michigan Medicine, Ann Arbor, Mich (M.M.L., N.D.P., C.C.); Institut für Röntgendiagnostik und Nuklearmedizin, Städtisches Klinikum Braunschweig, Braunschweig, Germany (P.W., M.P.); Klinik für Radiologie und Nuklearmedizin, Universitätsklinikum Magdeburg, Magdeburg, Germany (M.P., M.T.); Department of Radiology, Vall d’Hebrón University Hospital, Barcelona, Spain (X.S.C.); Department of Radiology, Medical College of Wisconsin, Milwaukee, Wis (S.B.W.); Department of Radiology, Tampa General Hospital, Tampa, Fla (C.D.); Department of Interventional Radiology, University of Chicago Pritzker School of Medicine, Chicago, Ill (O.A.); Departments of Biomedical Engineering, Radiology, and Neurosurgery, University of Michigan, Ann Arbor, Mich (Z.X.); Department of Radiology, University of Kansas Medical Center, Kansas City, Kan (Z.C.); Department of Interventional Radiology, Baptist Hospital of Miami, Miami, Fla (G.N.); Department of Biomedical Science, Humanitas University & Humanitas Clinical and Research Hospital IRCCS, Rozzano, Italy (G.T., L.S.); Department of Radiology, Freeman Hospital, Newcastle, United Kingdom (P.L.); Department of Diagnostic and Interventional Radiology, Leeds Teaching Hospital and Trust, West Yorkshire, United Kingdom (T.M.W.); and Department of Radiology, University of Wisconsin School of Medicine and Public Health, 600 Highland Ave, Madison, WI 53792 (T.J.Z.).

## Abstract

**Background:**

Histotripsy is a nonthermal, nonionizing, noninvasive, focused US technique that relies on cavitation for mechanical tissue breakdown at the focal point. Preclinical data have shown its safety and technical success in the ablation of liver tumors.

**Purpose:**

To evaluate the safety and technical success of histotripsy in destroying primary or metastatic liver tumors.

**Materials and Methods:**

The parallel United States and European Union and England #HOPE4LIVER trials were prospective, multicenter, single-arm studies. Eligible patients were recruited at 14 sites in Europe and the United States from January 2021 to July 2022. Up to three tumors smaller than 3 cm in size could be treated. CT or MRI and clinic visits were performed at 1 week or less preprocedure, at index-procedure, 36 hours or less postprocedure, and 30 days postprocedure. There were co-primary end points of technical success of tumor treatment and absence of procedure-related major complications within 30 days, with performance goals of greater than 70% and less than 25%, respectively. A two-sided 95% Wilson score CI was derived for each end point.

**Results:**

Forty-four participants (21 from the United States, 23 from the European Union or England; 22 female participants, 22 male participants; mean age, 64 years ± 12 [SD]) with 49 tumors were enrolled and treated. Eighteen participants (41%) had hepatocellular carcinoma and 26 (59%) had non–hepatocellular carcinoma liver metastases. The maximum pretreatment tumor diameter was 1.5 cm ± 0.6 and the maximum post-histotripsy treatment zone diameter was 3.6 cm ± 1.4. Technical success was observed in 42 of 44 treated tumors (95%; 95% CI: 84, 100) and procedure-related major complications were reported in three of 44 participants (7%; 95% CI: 2, 18), both meeting the performance goal.

**Conclusion:**

The #HOPE4LIVER trials met the co-primary end-point performance goals for technical success and the absence of procedure-related major complications, supporting early clinical adoption.

Clinical trial registration nos. NCT04572633, NCT04573881

Published under a CC BY 4.0 license.

*Supplemental material is available for this article.*

See also the editorial by Nezami and Georgiades in this issue.

## Graphical abstract

**Figure:**
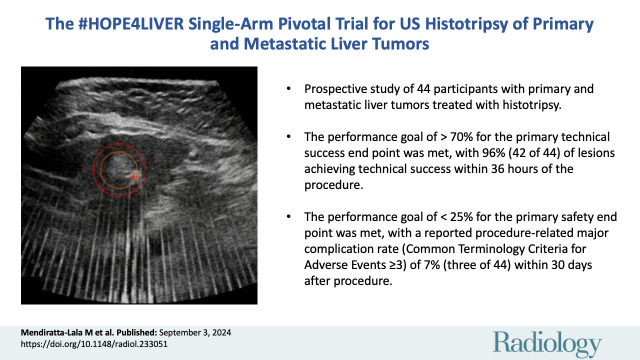

**Summary**

The pooled #HOPE4LIVER trials demonstrated the safety and technical success of the HistoSonics System for the destruction of liver tumors using histotripsy, a non-thermal, mechanical process of focused US.

**Key Results**

■ In a prospective study of 44 participants with primary and metastatic liver tumors treated with histotripsy, the performance goal of greater than 70% for the primary technical success end point was met, with 95% (42 of 44) of lesions achieving technical success within 36 hours of the procedure.
■ The performance goal of less than 25% for the primary safety end point was met, with a reported procedure-related major complication rate (Common Terminology Criteria for Adverse Events ≥3) of 7% (three of 44) within 30 days postprocedure.

## Introduction

Histotripsy is a noninvasive, nonthermal, and nonionizing focused US modality in which short, high-pressure ultrasound waves create local tissue pressure changes at a focal point, resulting in cavitation and mechanical tissue liquefaction that can be viewed in real time (1). Preclinical studies have shown that histotripsy can adequately treat malignant tumors in small animal models, create clinically relevant treatment zones in large animal models, and spare collagenous structures such as blood vessels and bile ducts due to the mechanical mechanism of tissue destruction (1). The THERESA study (2), a feasibility trial in hepatocellular carcinoma and hepatic metastases, demonstrated hepatic histotripsy could safely and effectively destroy a targeted tissue volume in humans.

Hepatocellular carcinoma is currently the fourth-leading cause of cancer-related mortality worldwide (3). Local-regional treatments, including thermal ablation, chemoembolization, bland embolization, radioembolization, and stereotactic body radiation therapy, are common treatment options for early-stage disease, according to the guidelines published by scientific societies (4,5). When technically feasible, these treatment options are often preferred in the earlier stages of hepatocellular carcinoma.

Around 50% of patients with colorectal cancer develop liver metastases. Local control by surgical resection prolongs overall survival (6), but only 10%–20% of patients are eligible for surgical resection because of the number and/or size of lesions or inadequate liver reserve (7). Therefore, when technically feasible for size, location, and number of lesions, local-regional treatments are recommended options (8–11).

When local-regional treatments are performed for hepatocellular carcinoma and metastatic disease with the appropriate indications and correct technique, outcomes are generally good. However, limitations include treatment failure due to local tumor progression from incomplete treatment or complications, including unexpected treatment of extra-tumoral liver parenchyma or adjacent organs, intra- or extrahepatic bleeding, and thermal or radiation injury. Treatment options like histotripsy, which overcome these limitations, could increase the ability to treat tumors in patients who may not have other options (12–14). Therefore, the parallel United States and European Union and United Kingdom #HOPE4LIVER pivotal trials with pre-established end points aimed to evaluate the technical success and safety of the HistoSonics System (HistoSonics) for the treatment of primary or metastatic tumors located in the liver.

## Materials and Methods

### Study Design

The primary analysis for the #HOPE4LIVER United States trial pooled the participants enrolled in #HOPE4LIVER United States (ClinicalTrials.gov NCT04572633) at eight sites and #HOPE4LIVER European Union and England (ClinicalTrials.gov NCT04573881) at six sites (Table S1). These single-arm, nonrandomized, prospective, pivotal trials evaluated the technical success and safety of the HistoSonics System for the treatment of primary or metastatic liver tumors. The trials were sponsored and financed by HistoSonics. Trial design was reviewed by the competent authorities for each European geography and the U.S. Food and Drug Administration, where the results were submitted as part of the de novo submission for marketing approval. The protocol was approved by each institutional review board or ethics committee, the U.S. Food and Drug Administration, or each country’s competent authority in the European Union and England. The #HOPE4LIVER European Union and England study design was previously described (15); #HOPE4LIVER United States was conducted using an identical protocol.

### Participant Selection

Patients who signed a written informed consent, met inclusion and exclusion criteria, and underwent histotripsy treatment were enrolled in the trial. The inclusion and exclusion criteria are described in Appendix S1.

### Histotripsy Device and Procedure

The HistoSonics System is designed for the noninvasive destruction of liver tumors using a nonthermal, mechanical process of focused ultrasound. A complete description of the device is included in Appendix S1. The device uses ultrasonic pulses (700 kHz) of less than 20 µsec in length at a low-duty cycle (>1%) to induce high-peak negative pressures (>10 mPa) and thus inertial acoustic cavitation at a focal point. This results in an area of mechanical tissue destruction of approximately 3 mm × 3 mm × 6 mm. The therapy transducer, which has a coaxially aligned diagnostic transducer for B-mode US targeting, is mounted to a robotic arm. Software controls allow the focal point to be moved through a prescribed volume of tissue, creating the treatment effect. Treatment depth was not recorded, though is limited by the natural focus of the therapy transducer with a maximum operating depth of 14–16 cm.

### Trial End Points

The co-primary end point of technical success was defined as the tumor treatment volume being greater than or equal to the targeted volume with complete tumor coverage, and evaluated at contrast-enhanced CT or MRI 36 hours or fewer postprocedure. The treated tissue was expected to immediately demonstrate no residual mass-like enhancement at follow-up imaging posttreatment (1). A lesion success performance goal of 70% was established from real-world evidence where a lower limit of greater than 70% for 95% CI, would indicate the technical success rate as higher than the performance goal at a one-sided *P* < .025.

The co-primary safety end point was defined as the absence of procedure-related major complications through 30 days postprocedure in a participant, with major complications defined as an adverse event and classified as Common Terminology Criteria for Adverse Events (version 5.0) grade 3 or higher (16). A safety performance goal of 25% was established from real-world evidence where an upper limit of the 95% CI less than 25% would indicate that the procedure-related major complication rate was lower than the performance goal at a one-sided *P* < .025 significance level. The trial was deemed successful if both primary end points were met.

A secondary end point was assessed via technique efficacy, defined as the lack of a nodular or mass-like area of enhancement within or along the edge of the treatment volume assessed at contrast-enhanced CT or MRI at 30 days postprocedure per standard guidelines (17). The secondary safety end point was the summarization of all adverse events reported within 30 days postprocedure.

### Primary Analysis Population

The primary analysis required an evaluable technical success outcome in 40 enrolled participants pooled from both trials. Due to the strict inclusion and exclusion criteria, a sample size of 40 participants was agreed on in consultation with the U.S. Food and Drug Administration during the study design. Subsequently, a performance goal was selected based on providing the U.S. Food and Drug Administration with reasonable assurance of the safety and technical success of the device for the indication being sought. Determination of sample size is described in Appendix S1. To assess 40 evaluable participants, the first 44 consecutive participants enrolled between January 2021 and July 2022 were analyzed.

### Independent Assessments

Core laboratory reviews of CT and MRI data were performed by two board-certified, fellowship-trained radiologists with more than 15 years of experience through Intrinsic Imaging using standardized guidelines (17) (Appendix S1). Adjudication of Common Terminology Criteria for Adverse Events coding and relatedness was performed by a clinical events committee by majority decision from three physicians with interventional radiology and/or oncology experience and who were not affiliated with the sponsor or institutions conducting the trial. A data safety monitoring board that included three physicians and a statistician, independent from the sponsor, provided ongoing oversight of the trials. Statistical analysis was performed by Quantics Biostatistics and Liu Associates Consulting.

### Statistical Analysis

A predefined statistical analysis plan established the statistical methods for the trial. Analyses were performed using statistical software (R, version 3.4.1 or later, R: A Language and Environment for Statistical Computing; and SAS, version 9.4 or later, SAS Institute) and validated as a diverse self-checking pair.

Categorical data are presented as numbers and percentages and numeric data are presented as means ± SDs. Technical success was assessed as the number of successfully treated lesions divided by the total number of treated lesions; the two-sided nonparametric 95% Wilson score CI was estimated by bootstrap sampling with a replacement method to account for potential within-participant lesion correlations. Participant was the bootstrap sampling unit, 1 000 000 iterations for bootstrap resampling were performed, and the bootstrap method was the bias-corrected and accelerated method. Per the study protocol, only those with complete imaging data were included in the technical success cohort. Primary safety was assessed as the participant incidence rate of procedure-related major complications and its two-sided 95% Wilson score CI on all enrolled participants. Secondary end points are presented descriptively with no predefined hypothesis tests. Details regarding the analyses performed can be found in Appendix S1.

## Results

### Participant Characteristics

**Figure 1:**
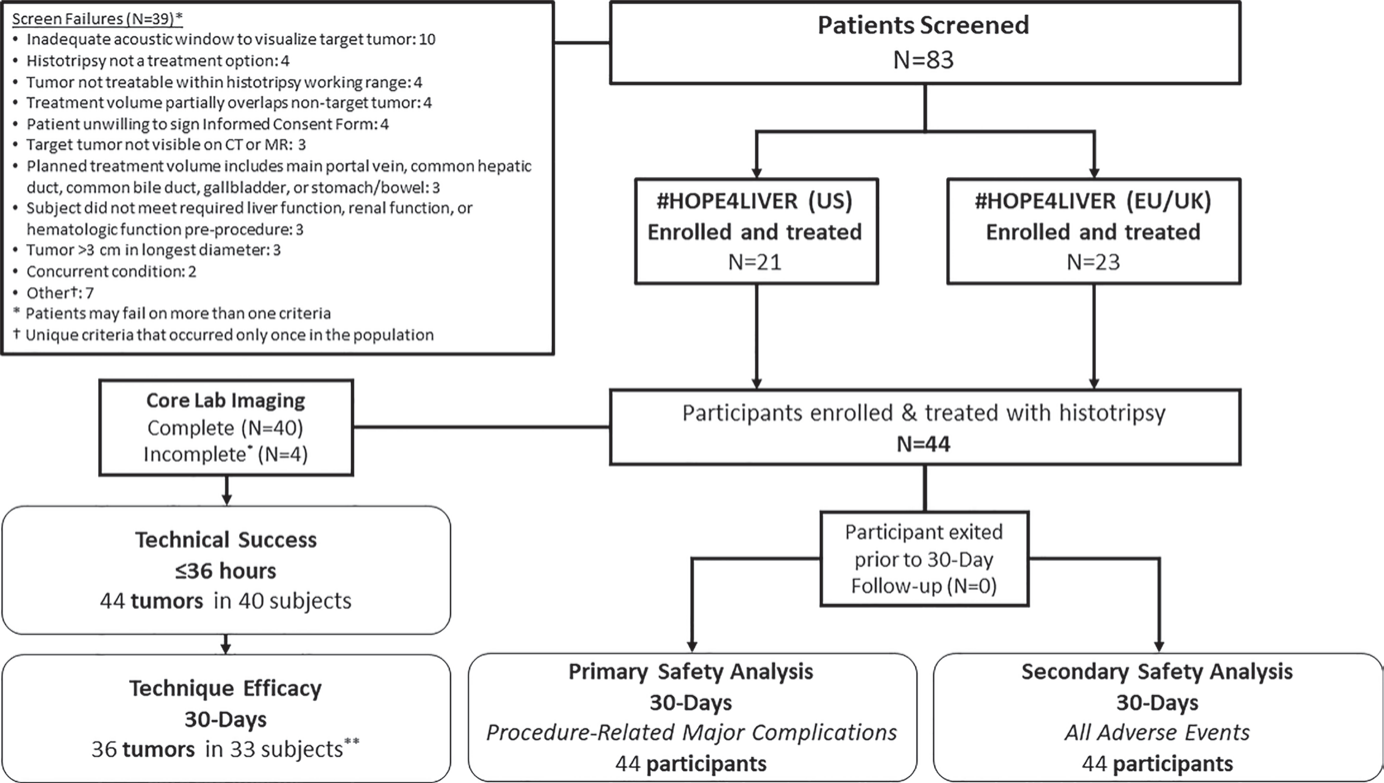
Study design flowchart. * = Incomplete due to poor image quality (three tumors in three participants) and lack of contrast (two tumors in one participant). ** = Six lesions (five participants) did not have 30-day imaging and the core laboratory could not assess technique efficacy in two lesions (two participants).

Of the 83 patients initially screened, 44 participants were enrolled and treated (21 participants from the United States and 23 participants from the European Union or England). Participant exclusion criteria are described in Figure 1. The mean participant age was 64 years ± 12 (SD) and 50% were female participants. The majority had Child-Pugh class A disease (86%). Eighteen participants (41%) were diagnosed with hepatocellular carcinoma. The remaining 26 participants (59%) had liver metastases from the colon (*five participants*, 19%), rectum (five *participants*, 19%), pancreas (five *participants*, 19%), and breast (four *participants*, 15%). The remaining seven participants had metastases from uveal melanoma (two *participants*, 8%), liver neuroendocrine (one *participant*, 4%), lung neuroendocrine (one *participant*, 4%), brain hemangiopericytoma (one *participant*; 4%), ovarian carcinoma (one *participant*, 4%), and bronchial carcinoma (one *participant*, 4%). A total of 49 tumors were treated. Thirty-nine participants (89%) had one tumor and five participants (11%) had two tumors treated during the procedure. Further participant characteristics are summarized in Table 1. Participants were followed through the 30-day visit.

**Table 1:**
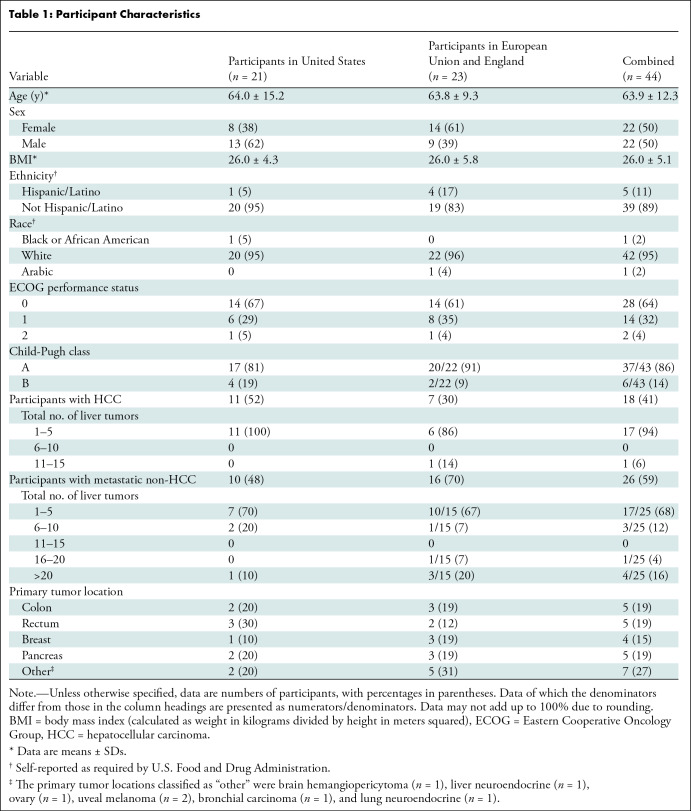
Participant Characteristics

### Histotripsy Treatment

**Figure 2:**
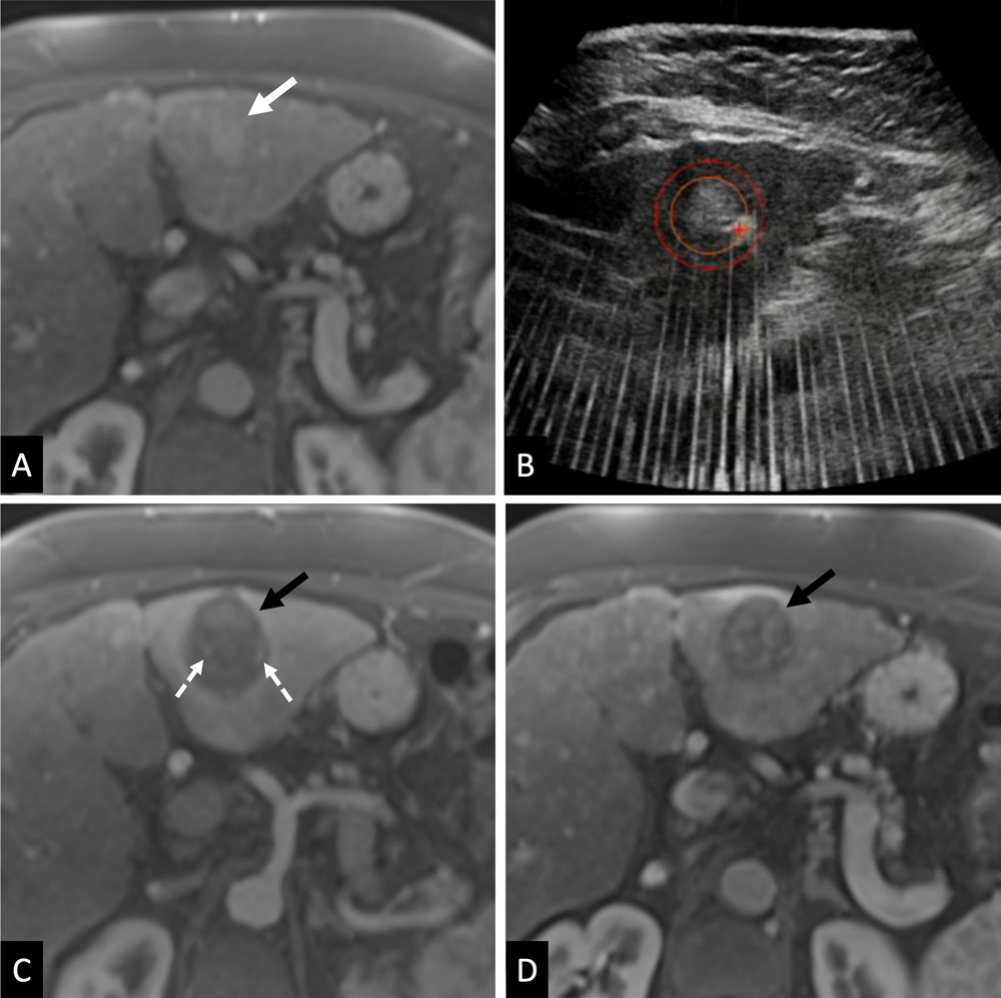
Images in a participant with a 2.3-cm hepatocellular carcinoma in segment III of the liver. **(A)** Preprocedure contrast-enhanced MRI scan in the late arterial phase shows hyperenhancing hepatocellular carcinoma (arrow). **(B)** Intraprocedural US image shows defined treatment volume; the orange circle outlines the hyperechoic targeted tumor and the red circle outlines the planned margin. The treatment effect is visible as an echogenic bubble cloud at the red crosshair, the focal point of the transducer. The robotic arm moves the focal point through the planned treatment volume to create the volumetric treatment. **(C)** Contrast-enhanced MRI scan obtained less than 36 hours postprocedure in the late arterial phase shows the nonenhancing treatment zone (black arrow). Note patent blood vessels (dashed white arrows). **(D)** Contrast-enhanced MRI scan obtained 30 days postprocedure in the late arterial phase shows partial involution of the treatment zone (arrow).

Histotripsy procedures were performed with general anesthesia. The mean histotripsy treatment time (from the start of automated treatment to completion for each tumor) was 34 minutes ± 25 (*n* = 46) with a total procedure duration of 221 minutes ± 64. Histotripsy was completed during a single procedure in all participants and no adjunctive ablation or resection was performed in any participant. Representative treatment is shown in Figure 2.

### End-Point Results

Complete baseline and postprocedure imaging assessment could not be performed by the core laboratory due to poor image quality (three tumors in three participants) and lack of contrast administration (two tumors in one participant); therefore, 44 tumors were included for evaluation of technical success. Treated tumors were in hepatic segments II (*n* = 6), III (*n* = 24), IV (*n* = 8), V (*n* = 3), and VI (*n* = 3). The maximum pretreatment tumor diameter was 1.5 cm ± 0.6 and the maximum post-histotripsy treatment zone diameter was 3.6 cm ± 1.4. The characteristics of the 44 tumors evaluated for technical success are summarized in Table 2 and summarized for all enrolled participants in Table S2.

**Table 2:**
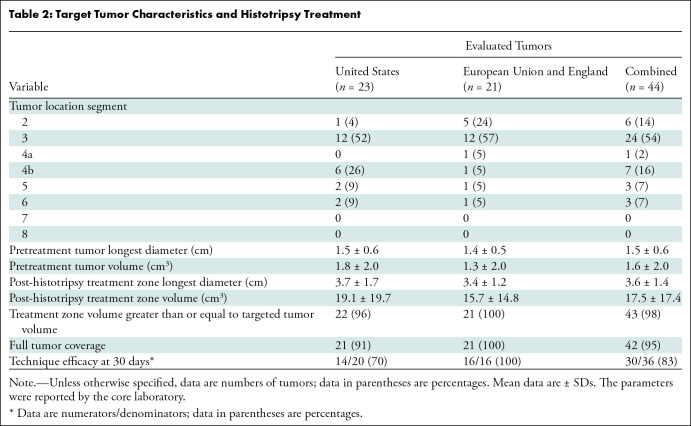
Target Tumor Characteristics and Histotripsy Treatment

**Figure 3:**
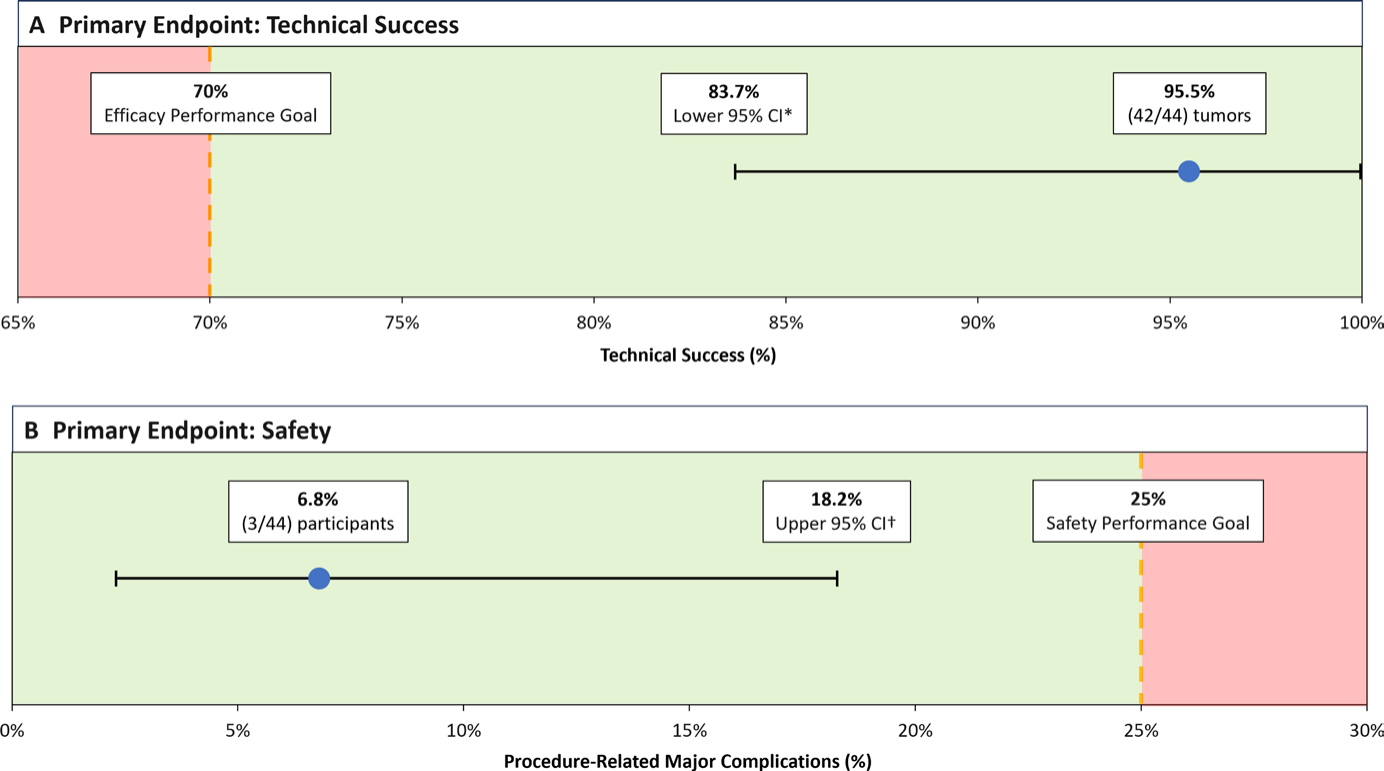
Graphs show primary end points of **(A)** technical success and **(B)** safety of procedure-related major complications. * = Estimated by bootstrap sampling with replacement method to account for potential within-subject lesion correlations. Subject was the bootstrap sampling unit, 1 000 000 iterations for bootstrap resampling were performed, and the bootstrap method was the bias-corrected and accelerated method. † = Two-sided 95% Wilson score CI.

The technical success end point was achieved in 42 of 44 tumors (95%; 95% CI: 84, 100) (Fig 3). The lower bound of 84% was greater than the predefined performance goal of 70%, indicating the null hypothesis was rejected and the technical success goal was met. Technical success on the participant level was performed as a sensitivity analysis assuming total correlation, choosing the worst outcome if multiple lesions were treated. Technical success was achieved in 38 of 40 participants (95%; 95% CI: 84, 99) (Table S3). Additionally, in a post hoc sensitivity analysis using exact CIs, technical success was achieved in 42 of 44 lesions (95%; 95% CI: 85, 99) (Table S4). The other predefined sensitivity analyses confirmed the result of the primary analysis (Tables S5–S7). The core laboratory imaging review determined that both tumors without technical success were not fully covered by the histotripsy treatment zone, a result of mistargeting, and one of the two treatment zones was also volumetrically smaller than the tumor volume. In participants assessed for technical success, core laboratory assessment of off-target damage, defined as any damage to liver tissue outside of the expected margin with “damage” represented by imaging changes versus baseline, was reported in six (14%) cases. One case (2%) was due to mistargeting where histotripsy was performed anterior to the tumor and five (11%) were perfusion changes contiguous to the treatment area.

**Figure 4:**
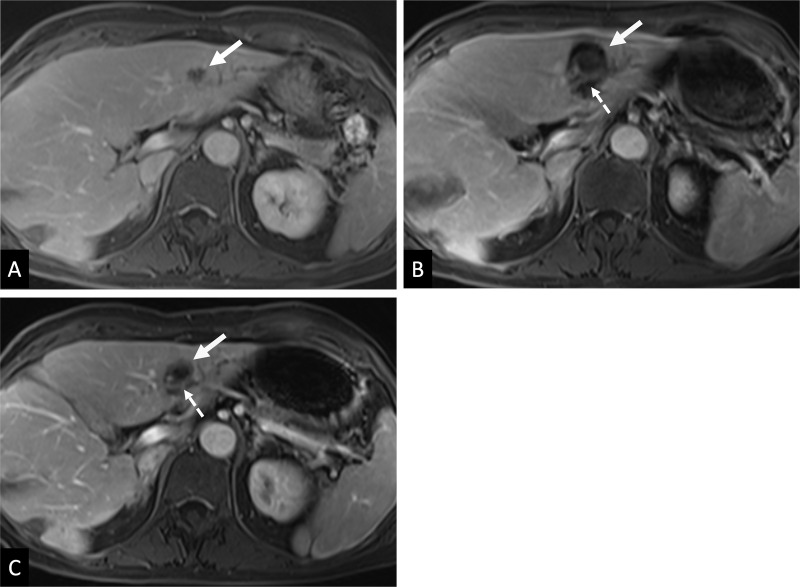
Images show a 1.3-cm metastatic colorectal cancer in segment III of the liver. **(A)** Preprocedure contrast-enhanced MRI scan shows tumor (arrow). **(B)** Contrast-enhanced MRI scan obtained fewer than 36 hours postprocedure with treatment zone (solid arrow) encompassing the site of the tumor and a margin. Patent blood vessel (dashed arrow) traverses the treatment zone. **(C)** Contrast-enhanced MRI scan obtained at 30 days postprocedure shows involution of the treatment zone (solid arrow) with maintained patent vessel (dashed arrow).

Technique efficacy at 30 days postprocedure was assessed in 36 lesions because six lesions did not have 30-day imaging and technique efficacy could not be assessed in two lesions by the core laboratory. Technique efficacy at 30 days was 83% (95% CI: 68, 92) and was achieved in 30 of 36 lesions. A representative MRI scan of histotripsy treatment at less than 36 hours and at 30 days is shown in Figure 4.

Index procedure–related major complications (primary safety failures) were reported in three of 44 participants (7%; 95% CI: 2, 18) (Fig 3). The upper bound of 18% was less than the predefined performance goal of 25%, indicating that the null hypothesis was rejected and the primary safety goal was met. Two participants experienced Common Terminology Criteria for Adverse Events grade 3 events (sepsis in a participant with a pre-existing indwelling biliary stent treated with antibiotics and pleuritic pain in a participant requiring 2-day admission and treatment with paracetamol) and one participant experienced a grade 5 event (hepatic failure on day 12 in a participant with innumerable breast primary metastatic lesions, which resulted in death 37 days postprocedure).

A total of 101 adverse events occurred within the first 30 days postprocedure and were assessed for secondary safety. Ninety-four adverse events (93.1%) were considered nonserious (Table S8). The most frequently reported (>5%) nonserious adverse events in the 44 participants were abdominal pain (10 participants, 23%), procedural pain (nine participants, 20%), pyrexia (six participants, 14%), fatigue (five participants, 11%), atelectasis (four participants, 9%), back pain (three participants, 7%), and anemia (three participants, 7%). There were seven adverse events classified as serious; three were described above as primary safety end-point failures. The remaining four were as follows: splenic hematoma; melena; procedural pain; and metastatic colorectal cancer, which was reported as “progression metastatic colorectal cancer” by the investigator (Table S9). No procedure-related bleeding events or deaths were reported in the first 30 days.

## Discussion

In the #HOPE4LIVER trial of histotripsy, a nonthermal, nonionizing, noninvasive focused ultrasound technique for treating liver tumors, the primary study end points were met, with a technical success rate of 95% (42 of 44 lesions) and a procedure-related major complication rate of 7% (three of 44 participants), demonstrating the ability of histotripsy to treat a targeted volume of liver tissue successfully and safely.

Mechanical tissue destruction induced by histotripsy has been extensively studied in preclinical models. The studies demonstrated histotripsy’s ability to spare critical collagenous structures such as large blood vessels and bile ducts and to induce effects on distant nontreated tumors; both are areas of potential therapeutic benefit (1). Ex vivo work first demonstrated the sparing of collagenous structures with direct histotripsy treatment, particularly when exposed to clinically relevant doses and dwell time (18). These findings have been confirmed using in vivo animal models and in human patients (2,19). Additionally, multiple rodent tumor models have demonstrated immune infiltration of histotripsy-treated tumors with evidence of abscopal tumor response in nontreated tumors (20–23). This response in nontreated tumors has also been noted in a pilot human trial (2). Given that tissue-sparing and nontarget tumor response were not directly evaluated in this study, the potential of these findings supports further exploratory work.

The technical success rate of 95% (42 of 44 lesions) achieved in this trial compares favorably with literature-reported rates of more established local treatments, including percutaneous radiofrequency ablation and microwave ablation, where rates in larger series have ranged from 48% to 95% across tumor types (11,24–30). The average 3.6-cm treatment zone for an average 1.5-cm tumor is encouraging because the technical success of any ablative modality is related to the achievement of a sufficiently thick, three-dimensional ablative margin (25,31). The technique efficacy at 30 days of 83% (30 of 36) achieved in a single procedure is like those achieved in the earliest reports of radiofrequency ablation (32) and may improve with procedural experience. Additionally, the standards used to evaluate local control in this study were adapted from ablation guidelines, where residual enhancing tissue at the periphery of the treatment zone is considered local tumor progression (17). However, because of the collagen-sparing effects of histotripsy, blood vessels can be seen within the treatment zone and classified as enhancing tissue at image analysis. Long-term follow-up of treatment zones is needed to determine a more accurate rate of local control. Of note, following histotripsy, local control may be more straightforward to evaluate because treatment zones undergo relatively rapid involution, with 89% volume involution at 2 months in a human trial of hepatic histotripsy (2).

Major complication rates are difficult to compare across studies due to varying grading systems. The 7% major complication rate experienced in this study is within reported ranges for local-regional treatment of liver tumors (ie, between 2% and 11% for the largest series of percutaneous radiofrequency ablation and microwave ablation) (9,10,23–26,33). Occurrence of infection after treatment in a participant with an indwelling biliary stent was not unexpected and has an increased rate of up to 50% in the setting of local treatment (34). There was a Common Terminology Criteria for Adverse Events grade 5 complication, which must be acknowledged. This occurred in a participant with metastatic disease burden in whom the planned treatment volume was achieved. Whereas the participant met inclusion criteria for hepatic function, their underlying liver function was likely tenuous. Although this trial included patients with advanced disease, close attention to underlying liver function is recommended in future patient selection for histotripsy procedures. Given the noninvasive nature of the procedure and the potential of vessel sparing, a lack of procedure-related bleeding complications was expected. Interestingly, prior studies in animals (35), including animals supratherapeutic on warfarin, have shown histotripsy to be safe with no evident bleeding complications even with anticoagulation.

The study had notable limitations. First, the small sample size and short-term postprocedure follow-up were limitations related to the nature of the study design. Larger patient populations are likely to be reported when patient enrollment expands beyond a clinical trial. Second, the technical success goal, although literature-based, was decided on with the U.S. Food and Drug Administration, based on an achievable patient enrollment goal, and was not intended to identify a difference from existing therapies. Long-term follow-up of the current trial participants is planned and is expected to be reported in the future. Third, given the lack of validated treatment-response criteria, the 30-day efficacy assessments were extrapolated from existing standards of treatment response despite the unique mechanism of cell destruction when using histotripsy. An improved understanding of imaging outcomes is likely to evolve in future studies. Fourth, the total procedure time was difficult to compare with that reported for existing therapies because investigators had no prior experience with this or similar externally delivered therapies. Finally, the patient population was not typical of those who undergo ablative therapies because many had stage IV metastatic disease. These somewhat limit the comparison of histotripsy with reported outcomes of other well-established local-regional treatments.

In conclusion, this pivotal study confirms the technical success and safety of histotripsy for the noninvasive mechanical destruction of liver tumors. Pending early clinical adoption, larger trials with longer follow-up in typical candidates for local-regional treatment will provide further outcome data to help define the role of this emerging technology.
